# Associations with other cancer-related biomarkers might contribute to poor outcomes in *RAS*-altered, younger patients with colorectal cancer

**DOI:** 10.1093/oncolo/oyae153

**Published:** 2024-06-17

**Authors:** Madappa N Kundranda, Ariane C Kemkes, Mark C Evans, Cynthia A Flannery, David W Hall, Jess R Hoag, Nishitha Therala, Snehal G Thakkar, Jean-Paul De La O

**Affiliations:** Banner MD Anderson Cancer Center, Gilbert, AZ, United States; Exact Sciences Corporation, Madison, WI, United States; Exact Sciences Corporation, Madison, WI, United States; Exact Sciences Corporation, Madison, WI, United States; Exact Sciences Corporation, Madison, WI, United States; Exact Sciences Corporation, Madison, WI, United States; Exact Sciences Corporation, Madison, WI, United States; Exact Sciences Corporation, Madison, WI, United States; Exact Sciences Corporation, Madison, WI, United States

**Keywords:** colorectal cancer, genetic associations, genomics, RAS, APC, RNF43, MSI-high

## Abstract

Colorectal cancer (CRC) is a common cancer in younger adults. In patients undergoing liver resection with *RAS*-altered CRCs, there is evidence suggesting younger patients have worse outcomes than older patients. To explain this pattern, differences in associations between *RAS* status and other cancer-related biomarkers in tumors from younger versus older patients with CRC were evaluated in a cohort of 925 patients with CRC, 277 (30.0%) of whom were ≤50 years old, and 454 (49.1%) who had *RAS*-altered tumors. For 3 biomarkers, *RNF43*, *APC*, and microsatellite instability (MSI), the association with *RAS* status was significantly modified by age after adjustment for multiple testing. Specifically, younger patients with *RAS*-altered tumors were more likely to be MSI-high, *RNF43* mutated, and *APC* wild type. These differences might contribute to the observed pattern of diminished survival in younger versus older patients with CRC with *RAS*-mutated tumors undergoing liver metastasis resection.

## Teaser

In patients undergoing liver resection with RAS-altered colorectal cancers (CRCs), there is evidence suggesting younger patients have worse outcomes than older patients. To explain this pattern, this study evaluated differences in associations between RAS status and other cancer-related biomarkers in tumors from younger versus older patients with CRC.

## Introduction

Among younger adults, defined using a cutoff of 50 years, CRC represents the second most common cancer diagnosis and the third leading cause of cancer death.^1^ CRC in younger adults is characterized by unique clinical, genetic, and epigenetic characteristics, which may affect overall survival compared to CRC diagnosed among older individuals.^2^ For example, there are notable differences between younger and older patients in the relative frequency of altered driver^3^ and germline variants.^4^

Outcomes could differ between younger and older patients with CRC even when genetic alterations are similar. A report of patients with CRC who had undergone surgical resection for liver metastasis noted poorer outcomes if they harbored *RAS* alterations; but the effect appeared to be more pronounced in younger patients.^5^ While age per se may be an independent risk factor, the effect could also be attributed to alterations in other cancer-related biomarkers that are associated with *RAS* alterations in younger patients. To address whether other altered biomarkers might contribute to the pattern of worse outcomes in *RAS*-altered younger patients versus older patients, we examined the distribution of other altered biomarkers by *RAS* status and age.

## Methods

Tumor samples from patients with metastatic CRC profiled with the tumor-normal, whole-exome, whole-transcriptome OncoExTra assay between April 2018 and July 2022 were included. We focused on DNA alterations deemed to be clinically actionable, defined as: (1) pathogenic within the cancer genome, (2) associated with an FDA-approved therapy, (3) an eligibility criterion for clinical trial enrollment, or (4) deemed significant based on recent literature review.

Associations between *RAS* status and other biomarker alterations were examined by age group (≤50 years vs >50 years). Differences in associations between patients ≤50 years versus >50 years were tested using the Breslow-Day (B-D) statistic, which tests the homogeneity of odds ratios (ORs). We restricted our analysis to biomarkers that were altered in at least 10 patients to maintain minimal sample sizes for statistical testing and clinical relevance, and adjusted *P*-values for the false discovery rate, to give *Q*-values, to account for multiple testing. The significance threshold was set to 0.05. All analyses were performed using SAS software (version 9.4, SAS Institute, Cary, NC).

## Results

There were 925 metastatic CRC patient samples analyzed; 454 (49.1%) were *RAS* altered and 471 (50.9%) were *RAS* wild type. The majority of *RAS* alterations were at *KRAS* (*n* = 419, 92.3%), and the remainder were at *NRAS* (*n* = 35, 7.7%). No *HRAS* alterations were seen. Most patients were age >50 years (*n* = 648, 70.0%), and *RAS* status did not differ by age (*RAS* altered 48.4% ≤50 years vs 49.4% >50 years, *P* = .78). We observed alterations at 169 non-*RAS* genes, and 49 of these were altered in 10 or more tumor samples. Tumor mutational burden (TMB) was high (≥10 mut/Mb) in 82 samples (8.9%) and microsatellite instability (MSI) was high in 60 (6.5%) samples; all samples that showed MSI-high were also TMB-high. A summary of these 51 altered biomarkers (49 genes, TMB, and MSI) and their associations with *RAS* status by age are provided in Supplementary Table S1.

The association with *RAS* status was significantly modified by age after adjustment for multiple testing for 3 biomarkers (*RNF43*, MSI-High, and *APC*; Table 1). Both *RNF43*-altered and MSI-high biomarkers were more prevalent in *RAS*-altered younger patients. A total of 8.2% of patients ≤50 years had both *RAS* and *RNF43* alterations, compared to 2.8% of patients >50 years. Among patients ≤50 years, a positive yet nonsignificant association was observed between *RNF43*-altered status and *RAS*-altered status (OR: 2.47, 95% CI, 0.83-7.30, *P* = .12); however, among patients >50 years, the odds of being *RAS* altered were significantly lower for *RNF43*-altered versus *RNF43-*wild type patients (OR: 0.24, 95% CI, 0.11-0.51, *P* < .001; B-D test, *P* < .001). A similar pattern was observed for the association between *RAS* status and MSI-high by age (B-D test, *P* = .002). In contrast, *APC* alterations were less frequent in *RAS*-altered younger patients (67.9%) compared to older patients (82.8%) or, equivalently, unaltered (wild type) *APC* was more frequent. Among patients ≤50 years there was a nonsignificant negative association between *APC*-altered status and *RAS*-altered status (OR: 0.74, 95% CI, 0.44-1.24, *P* = .25); however, among patients >50 years, the odds of being *RAS* altered was significantly higher for *APC* altered versus *APC* wild type (OR: 1.96, 95% CI, 1.35-2.86, *P* < .001; B-D test, *P* = .003).

**Table 1.** Distribution of biomarkers exhibiting a significantly different association with *RAS* status in younger versus older adults, as determined by the B-D test, after correcting for the false discovery rate.

Biomarker	Age ≤ 50 years (*n* = 277)		Age > 50 years (*n* = 648)		B-D *P*-value	B-D *Q*-value
	*RAS* ^WT^ (*n* = 143)	*RAS* ^alt^ (*n* = 134)	*RAS* ^WT^ (*n* = 328)	*RAS* ^alt^ (*n* = 320)		
*RNF43* ^WT^ (*n* = 865)	138	123	293	311		
*RNF43* ^alt^ (*n* = 60)	5	11	35	9		
	OR = 2.47 (CI, 0.83-7.30)		OR = 0.24 (**CI, 0.11-0.51**)		<.001	.012
Not MSI-High (*n* = 865)	138	123	296	308		
MSI-High (*n* = 60)	5	11	32	12		
	OR = 2.47 (CI, 0.83-7.30)		OR = 0.36 (**CI, 0.18-0.71**)		.002	.048
*APC* ^WT^ (*n* = 230)	37	43	95	55		
*APC* ^alt^ (*n* = 695)	106	91	233	265		
	OR = 0.74 (CI, 0.44-1.24)		OR = 1.96 (**CI, 1.35-2.86**)		.003	.048

Within each age group, for each biomarker the OR for an association and its 95% CI are shown; CIs that do not overlap 1.0 are bolded.

Abbreviations: B-D, Breslow-Day; WT, wild type (no somatic mutation); alt, altered; OR, odds ratio.

## Conclusions

In this study, we discovered that *RNF43,* when altered, *APC,* when wild type, and MSI-high appear to be more prevalent in *RAS*-altered tumors from younger patients with CRC. Could such a finding help to explain the poorer prognosis observed in *RAS*-altered younger patients compared to RAS-altered older patients undergoing liver resection?

There is evidence that all 3 of these biomarkers are prognostic for outcome in patients with CRC. Specifically, *APC*-wild type status is associated with poor response to chemotherapy, resulting in shorter overall survival.^6^ While MSI-high CRC tumors generally have more favorable outcomes, they are resistant to chemotherapy.^7^ Finally, patients with CRC with *RNF43*-altered tumors have poorer relapse-free survival^8^ and worse overall survival.^9^ Thus, it is possible that the associations observed, with biomarkers prognostic for poor outcome overrepresented in *RAS*-altered younger patients, could contribute to the observed pattern.

However, the frequency of *APC* wild type, *RNF43* altered, and MSI in younger patients with *RAS*-mutated tumors is only 5%-15% greater than in older patients, which may be insufficient to fully explain the observed 20% overall survival hazard ratio difference,^5^ even if the effects of these biomarkers on survival were substantial. It would be informative to examine the clinical outcomes for younger patients with CRC with *RAS*-mutated tumors who are *APC* wild type, *RNF43* altered, or have MSI tumors.

Nevertheless, our findings suggest that higher-order associations among biomarkers, *RAS* status and age are present in CRC tumors and may thus be a contributing factor to the apparent difference in outcome between *RAS*-altered younger and older patients with CRC after liver resection. Such higher-order genetic associations suggest that tumor profiling should be comprehensive, assaying multiple genes, as focusing on one or a few genes may fail to identify important prognostic biomarkers that could help to inform therapy decisions. The prevalence of these multibiomarker associations that differ by age of onset should be further investigated in CRC and other cancers.

Limitations of our study include the moderate sample size, and the lack of clinical data to confirm the *RAS* status and age interaction on outcomes following liver resection in this cohort.

## Supplementary material

Supplementary material is available at *The Oncologist* online.

oyae153_suppl_Supplementary_Table

## Data Availability

The data underlying this article are available in the article and in Supplementary Table S1.
