# Supplementary material for: Associations with other cancer-related biomarkers might contribute to poor outcomes in RAS-altered, younger patients with colorectal cancer
Source: Oncologist. 2024 Jun 17;29(9):e1228–30. doi: 10.1093/oncolo/oyae153 (PMC11379647; doi:10.1093/oncolo/oyae153)
Supplement: oyae153_suppl_Supplementary_Table [file oyae153_suppl_supplementary_table.pdf]

Supplementary Table 1:
Biomarkers present in tumor samples of at least 10 of 925 patients with colorectal cancer. For each biomarker, the numbers of *RAS* mutant and *RAS* wild type patients within each age category are shown. Within each age category, for each gene, the odds ratio for an association between *RAS* status (mutant versus wild type) and biomarker status (altered versus not) is shown, along with the 95% confidence intervals (CIs); CIs that do not overlap 1.0 are bolded. Also shown for each biomarker are the p- and q-values for the Breslow-Day tests of a significant difference between the odds ratios between age groups, indicative of a different association between *RAS* status (mutant versus wild type, WT) and biomarker status (altered versus unaltered) between the two age groups. The first three biomarkers listed are significant after correction for multiple tests (q ≤ 0.05). The remaining biomarkers are in alphabetic order.

		Age ≤50 years				Age >50 years					
**Biomarker**	**N**	***RAS* mutant (N=134)**	***RAS* WT (N=143)**	**OR**	**95% CI**	***RAS* mutant (N=320)**	***RAS* WT (N=328)**	**OR**	**95% CI**	**Breslow-Day Test p-value**	**FDR q-value**
APC	695	91 (67.9%)	106 (74.1%)	0.74	0.44-1.24	265 (82.8%)	233 (71.0%)	1.96	1.35-2.86	**0.003**	**0.048**
MSI-High	60	11 (8.2%)	5 (3.5%)	2.47	0.83-7.30	12 (3.8%)	32 (9.8%)	0.36	**0.18-0.71**	**0.002**	**0.048**
RNF43	60	11 (8.2%)	5 (3.5%)	2.47	0.83-7.30	9 (2.8%)	35 (10.7%)	0.24	**0.11-0.51**	**<0.001**	**0.012**
ARID1A	64	13 (9.7%)	9 (6.3%)	1.60	0.66-3.87	16 (5.0%)	26 (7.9%)	0.61	0.32-1.16	0.082	0.23
ARID2	20	3 (2.2%)	4 (2.8%)	0.80	0.17-3.62	4 (1.3%)	9 (2.7%)	0.45	0.14-1.47	0.56	0.75
ASXL1	37	9 (6.7%)	5 (3.5%)	1.99	0.65-6.09	11 (3.4%)	12 (3.7%)	0.94	0.41-2.16	0.29	0.56
ATM	45	9 (6.7%)	6 (4.2%)	1.64	0.57-4.75	20 (6.3%)	10 (3.0%)	2.12	0.98-4.60	0.70	0.87
AXIN2	24	3 (2.2%)	2 (1.4%)	1.61	0.27-9.82	10 (3.1%)	9 (2.7%)	1.14	0.46-2.85	0.74	0.89
BLM	16	3 (2.2%)	2 (1.4%)	1.61	0.27-9.82	2 (0.6%)	9 (2.7%)	0.22	0.05-1.04	0.085	0.23
BRAF	69	3 (2.2%)	17 (11.9%)	0.17	0.05-0.59	5 (1.6%)	44 (13.4%)	0.10	**0.04-0.26**	0.52	0.75
BRCA2	41	8 (6.0%)	5 (3.5%)	1.75	0.56-5.50	8 (2.5%)	20 (6.1%)	0.39	**0.17-0.91**	**0.034**	0.23
CCND2	15	2 (1.5%)	1 (0.7%)	2.15	0.19-24.01	7 (2.2%)	5 (1.5%)	1.44	0.45-4.60	0.77	0.89
CDKN2A	11	0 (0.0%)	2 (1.4%)	NA	-	6 (1.9%)	3 (0.9%)	2.07	0.51-8.35	0.090	0.23
CREBBP	16	1 (0.7%)	3 (2.1%)	0.35	0.04-3.42	5 (1.6%)	7 (2.1%)	0.73	0.23-2.32	0.57	0.75
CTNNB1	27	8 (6.0%)	5 (3.5%)	1.75	0.56-5.50	8 (2.5%)	6 (1.8%)	1.38	0.47-4.01	0.76	0.89
EP300	10	2 (1.5%)	3 (2.1%)	0.71	0.12-4.30	1 (0.3%)	4 (1.2%)	0.25	0.03-2.28	0.47	0.74
ERBB2	32	5 (3.7%)	3 (2.1%)	1.81	0.42-7.72	5 (1.6%)	19 (5.8%)	0.26	**0.10-0.70**	**0.022**	0.23
ERBB3	16	4 (3.0%)	1 (0.7%)	4.37	0.48-39.60	7 (2.2%)	4 (1.2%)	1.81	0.53-6.25	0.49	0.74
ERCC5	10	1 (0.7%)	2 (1.4%)	0.53	0.05-5.91	1 (0.3%)	6 (1.8%)	0.17	0.02-1.41	0.47	0.74
FANCM	14	1 (0.7%)	4 (2.8%)	0.26	0.03-2.37	2 (0.6%)	7 (2.1%)	0.29	0.06-1.40	0.94	0.98
FBXW7	96	19 (14.2%)	10 (7.0%)	2.20	0.98-4.92	43 (13.4%)	24 (7.3%)	1.97	1.16-3.32	0.82	0.91
GNAS	20	3 (2.2%)	1 (0.7%)	3.25	0.33-31.65	11 (3.4%)	5 (1.5%)	2.30	0.79-6.69	0.79	0.89

Biomarker	N	Age ≤50 years				Age >50 years				Breslow-Day Test p-value	FDR q-value
		RAS mutant (N=134)	*RAS* WT (N=143)	OR	95% CI	*RAS* mutant (N=320)	*RAS* WT (N=328)	OR	95% CI		
KDM6A	13	2 (1.5%)	2 (1.4%)	1.07	0.15-7.69	6 (1.9%)	3 (0.9%)	2.07	0.51-8.35	0.59	0.75
MAP2K1	14	1 (0.7%)	3 (2.1%)	0.35	0.04-3.42	1 (0.3%)	9 (2.7%)	0.11	0.01-0.88	0.45	0.74
MAP2K4	18	0 (0.0%)	6 (4.2%)	NA	-	3 (0.9%)	9 (2.7%)	0.34	0.09-1.25	0.18	0.42
MLH1	16	4 (3.0%)	2 (1.4%)	2.17	0.39-12.04	2 (0.6%)	8 (2.4%)	0.25	0.05-1.19	0.055	0.23
MLH3	12	0 (0.0%)	2 (1.4%)	2.73	0.52-14.33	0 (0.0%)	5 (1.5%)	NA	-	0.012	0.16
MSH2	10	2 (1.5%)	1 (0.7%)	2.15	0.19-24.01	1 (0.3%)	6 (1.8%)	0.17	0.02-1.41	0.090	0.23
MSH3	31	5 (3.7%)	4 (2.8%)	1.35	0.35-5.13	5 (1.6%)	17 (5.2%)	0.29	**0.11-0.80**	0.064	0.23
MSH6	34	7 (5.2%)	3 (2.1%)	2.57	0.65-10.16	9 (2.8%)	15 (4.6%)	0.60	0.26-1.40	0.069	0.23
MTOR	11	0 (0.0%)	3 (2.1%)	NA	-	2 (0.6%)	6 (1.8%)	0.34	0.07-1.68	0.35	0.61
MYC	20	0 (0.0%)	5 (3.5%)	NA	-	8 (2.5%)	7 (2.1%)	1.18	0.42-3.28	**0.036**	0.23
NF1	18	1 (0.7%)	5 (3.5%)	0.21	0.02-1.80	2 (0.6%)	10 (3.0%)	0.20	**0.04-0.92**	0.98	0.99
PBRM1	22	2 (1.5%)	6 (4.2%)	0.35	0.07-1.74	7 (2.2%)	7 (2.1%)	1.03	0.36-2.96	0.26	0.53
PIK3CA	166	33 (24.6%)	17 (11.9%)	2.42	1.28-4.60	72 (22.5%)	44 (13.4%)	1.87	1.24-2.83	0.51	0.75
PIK3R1	29	1 (0.7%)	5 (3.5%)	0.21	0.02-1.80	13 (4.1%)	10 (3.0%)	1.35	0.58-3.12	0.086	0.23
POLD1	12	3 (2.2%)	0 (0.0%)	NA	-	3 (0.9%)	6 (1.8%)	0.51	0.13-2.05	**0.041**	0.23
POLE	19	0 (0.0%)	5 (3.5%)	NA	-	5 (1.6%)	9 (2.7%)	0.56	0.19-1.70	0.12	0.30
PRKDC	24	4 (3.0%)	3 (2.1%)	1.44	0.32-6.54	6 (1.9%)	11 (3.4%)	0.55	0.20-1.51	0.30	0.56
PTCH1	14	3 (2.2%)	2 (1.4%)	1.61	0.27-9.82	1 (0.3%)	8 (2.4%)	0.13	0.02-1.01	**0.047**	0.23
PTEN	58	6 (4.5%)	5 (3.5%)	1.29	0.39-4.34	22 (6.9%)	25 (7.6%)	0.89	0.49-1.62	0.59	0.75
RAD50	19	5 (3.7%)	2 (1.4%)	2.73	0.52-14.33	5 (1.6%)	7 (2.1%)	0.73	0.23-2.32	0.19	0.42
RASA1	11	0 (0.0%)	4 (2.8%)	NA	-	2 (0.6%)	5 (1.5%)	0.41	0.08-2.11	0.24	0.51
RB1	10	1 (0.7%)	4 (2.8%)	0.26	0.03-2.37	1 (0.3%)	4 (1.2%)	0.25	0.03-2.28	0.99	0.99
SETD2	12	4 (3.0%)	2 (1.4%)	2.17	0.39-12.04	1 (0.3%)	5 (1.5%)	0.20	0.02-1.74	0.072	0.23
SMARCA4	16	2 (1.5%)	3 (2.1%)	0.71	0.12-4.30	4 (1.3%)	7 (2.1%)	0.58	0.17-2.00	0.86	0.93
TERT	13	2 (1.5%)	2 (1.4%)	1.07	0.15-7.69	7 (2.2%)	2 (0.6%)	3.65	0.75-17.68	0.33	0.60
TMB-High	82	12 (9.0%)	10 (7.0%)	1.31	0.55-3.14	18 (5.6%)	42 (12.8%)	0.41	**0.23-0.72**	**0.026**	0.23
TP53	696	82 (61.2%)	123 (86.0%)	0.26	0.14-0.46	223 (69.7%)	268 (81.7%)	0.51	**0.36-0.74**	**0.048**	0.23
TSC2	13	1 (0.7%)	2 (1.4%)	0.53	0.05-5.91	3 (0.9%)	7 (2.1%)	0.43	0.11-1.69	0.89	0.94
WRN	11	0 (0.0%)	2 (1.4%)	NA	-	6 (1.9%)	3 (0.9%)	2.07	0.51-8.35	0.090	0.23
